# Developmental changes in gene expression and enzyme activities of anabolic and catabolic enzymes for storage carbohydrates in the honeybee, *Apis mellifera*

**DOI:** 10.1007/s00040-018-0648-1

**Published:** 2018-07-13

**Authors:** E. Łopieńska-Biernat, K. Żółtowska, E. A. Zaobidna, M. Dmitryjuk, B. Bąk

**Affiliations:** 1 Department of Biochemistry, Faculty of Biology and Biotechnology, University Warmia and Mazury in Olsztyn, Oczapowskiego 1A, 10-719 Olsztyn, Poland; 2 Department of Apiculture, Faculty of Animal Bioengineering, University Warmia and Mazury in Olsztyn, Słoneczna 48, 10-957 Olsztyn, Poland

**Keywords:** *Apis mellifera*, Development, Enzymes, Gene expression, Glycogen, Trehalose

## Abstract

Glycogen and trehalose are important sources of energy in insects. The expression of genes encoding the key metabolic enzymes—glycogen synthase (GS), glycogen phosphorylase (GP), trehalose-6-phosphate synthase (TPS-1), soluble trehalase (Tre-1) and membrane-bound trehalase (Tre-2)—was analyzed in 12 developmental stages of *Apis mellifera* worker brood. The content of GS and GP proteins, TPS activity, total trehalase activity, and the activity of Tre-1 and Tre-2 were determined. Transcript quantity was not always correlated with the content of the encoded GS or GP protein. The correlation was higher for GS (*r* = 0.797) than GP (*r* = 0.651). The expression of the glycogen synthase gene (*gs*) and the glycogen phosphorylase gene (*gp*) was high in 4- and 7-day-old larvae and in pupae, excluding the last pupal stage. The expression of the *tps-1* gene was highest in the mid-pupal stage and contributed to higher enzyme activity in that stage. The expression of the *tre-1* gene was higher than the expression of the *tre-2* gene throughout development. In newly hatched workers, the expression of genes encoding catabolic enzymes of both carbohydrates, *gp* and *tre-1*, was higher than the expression of genes encoding anabolic enzymes. The results of this study suggest that sugar metabolism genes have somewhat different control mechanisms during larval development and metamorphosis.

**Electronic supplementary material:**

The online version of this article (10.1007/s00040-018-0648-1) contains supplementary material, which is available to authorized users.

## Introduction

Like other holometabolic insects, honeybee (*Apis mellifera*) development consists of four phases: egg, larval, pupal and adult. The development of honeybee brood may be also divided into two periods: in uncapped and in capped cells (from 8–9 days to adult emergence). During development of honeybee, profound morphological, anatomical (Rembold et al. 1980; Michelette and Soares 1993), biochemical and nutritional changes take place (Hrassingg and Crailshlem 2005). The larval phase is mainly a period of feeding and growth. During that time, the body weight of worker larvae increases 821-fold (Schmolz et al. 2005). Changes in food composition are observed on the fourth day of development of uncapped workers, and they determine also the worker phenotype. Worker larvae accumulate energy reserves which are used up by the capped brood in starvation mode (Winston 1987). In capped cell, individuals undergo metamorphosis, which means deep reorganization of larval structures and formation of imaginal structures. This process needs a lot of energy (Winston 1987).

The maintenance of energy homeostasis is the prerequisite for survival in all living organisms. Glucose is a universal source of energy for most organisms. Glucose can be derived from food or endogenous metabolic processes such as gluconeogenesis or breakdown of storage carbohydrates as glycogen and trehalose. Glycogen, a polymer of many glucose residues, is a common storage polysaccharide in all animals. Trehalose, a nonreducing disaccharide of glucose, is less widely distributed among animals. It is absent in vertebrates, but it is a very important sugar for invertebrates (Argüelles 2014). Both sugars are the most important carbohydrate reserves in insects. They are synthesized when the uptake of carbohydrates is greater than what is immediately required. Both can easily be converted to glucose when the reserves need to be mobilized, for example, during the flight (Neukirch 1982; Dulta and Verma 1989; Suarez 2000). Insects produce and store glycogen and trehalose mainly in fat bodies as well as in flight muscles, ovaries, tracheae and Malpighian tubules (Tang et al. 2012). Similarly, to other sugars, glycogen and trehalose levels are determined by the type of food, metabolic rate and developmental stage of insect (Blatt and Roces 2001; Tang et al. 2010). Glycogen is the predominant sugar in all developmental stages of *Apis mellifera*, and it accounts for 66% of total sugars in newly emerged workers to 77% in uncapped 4- and 6-day-old larvae. Trehalose accounts for 9.6–16.4% of total soluble sugars (Farjan et al. 2015). Level of glycogen and trehalose stores is important for honeybees in winter because they do not enter diapauses. Although metabolism of overwintering honeybees is lower than in bees during the summer, because they need sugars as a source of energy for the flight muscles to generate heat and maintain an appropriate temperature in the hive, which contributes to the survival of colony (Panzenböck and Crailsheim 1997; Stabentheiner et al. 2003).

Glycogen metabolism is similar in various organisms from bacteria (Wilson et al. 2010) to humans (Adeva-Andany et al. 2016). In all of them, glycogen metabolism requires two enzymes—glycogen synthase (EC 2.4.1.11) for synthesis and glycogen phosphorylase (EC 2.4.1.1) for breakdown. Both enzymes are hormonally controlled and play a key role in the metabolic balance of glycogen. Glycogen synthase (GS) catalyzes glycogen synthesis by donating glucose units from UDP-glucose to the primer (Chang et al. 2007). According to Vardanis (1967), GS differs in honeybee larvae and in mammals. In bees, it forms species-specific complexes with glycogen and occurs exclusively as glycogen synthase that is independent of G-6-P (GS-I). Glycogen phosphorylase (GP) releases glucose-1-phosphate (G-1-P) from glycogen, which is involved in the glycolytic pathway. In the following reaction, G-1-P is transformed into glucose 6-phosphate (G-6-P), a metabolite that connects synthesis and breakdown of storage insects’ sugars, both glycogen and trehalose (Klowden 2007; Tang et al. 2012). Glycogen can be broken down also by the hydrolytic pathway involving α-amylase and glucoamylase, but in bees the phosphorylation pathway involving GP is much stronger than the hydrolytic one (Żółtowska et al. 2012).

Trehalose plays in the hemolymph of insects a similar role as glucose in vertebrate blood. This non-reducing sugar (1,1′-α,d-glucopyranosyl-α,d glucopyranose) is characterized by low reactivity, and it can accumulate in the hemolymph of many insects at higher concentration (50–100 mM) than glucose without delivering toxic effects. Due to its molecular configuration, trehalose also protects and stabilizes cell membranes and proteins, and it protects cells against environmental stressors (Elbein et al. 2003). Trehalose concentration is determined by the activity of the key enzymes of trehalose metabolism. Two of them are linked with trehalose synthesis: trehalose-6-phosphate synthase (TPS, EC 2.4.1.15) which catalyzes the synthesis of trehalose-6-phosphate (T-6-P) from UDP-glucose and G-6-P, and trehalose-6-phosphate phosphatase (TPP, EC 3.1.3.12) which releases free trehalose. The breakdown of trehalose is catalyzed by a specific α-glucosidase—trehalase (Tre, EC 3.2.1.28). Trehalase hydrolyzes the α-1,1-glycosidic bond of trehalose to release two glucose molecules.

TPS genes have been cloned in many living organisms, including insects. Their genomic sequence is indicative of the conserved nature of the enzyme’s functional domains (see Tang et al. 2010; Kern et al. 2012). This observation led to the identification of the T-6-P synthase gene in the honeybee genome (Kunieda et al. 2006), but there is still lack of knowledge about structure and biochemical characteristic of this enzyme in honeybees. More is known about trehalase in bees. In *A. mellifera* and other insects (Tang et al. 2008; Tatun et al. 2008; Gu et al. 2009; Xie et al. 2013), this enzyme occurs in two forms as soluble trehalase and membrane-bound trehalase (Lefebvre and Huber 1970; Lee et al. 2007). In insects, differences in the expression of genes encoding both trehalases are determined by sex, developmental stage and tissue (Tang et al. 2008; Tatun et al. 2008; Xie et al. 2013). The relevant differences have not been studied in bees.

The expression of energy metabolism genes is altered during the development of insect, which has been observed in *Spodoptera exigua* (Tang et al. 2008, 2010, 2012). These processes have not been investigated in honeybees although their metabolism is based mainly on carbohydrates (Panzenböck and Crailsheim 1997). In our previous work, we studied the sugar metabolism only by analysis of the content of carbohydrates and the activities of their degradation enzymes during the honeybee development (Żółtowska et al. 2012; Farjan et al. 2015), but there is lack of knowledge about developmental changes in the enzymes’ activity (GS and TPS) that synthesize the key carbohydrates in the brood of honeybees. The aim of this study was to analyze and provide some connections between changes in gene expression of the key metabolic enzymes and trehalose and glycogen metabolism in different developmental stages of honeybees. This information is vital for describing saccharide metabolism in bees and expanding our knowledge about the biochemical nature of physiological processes during the post-embryonic development of these insects.

## Materials and methods

### Materials

The study was performed on the brood and newly emerged workers of the honeybee *A. mellifera*. The experimental material was collected in May 2012, from three bee colonies in a private apiary near Orneta, Poland. Honeycombs were wrapped in moist towels to maintain the appropriate moisture. Shortly after arrival at the laboratory, the brood was very carefully isolated from honeycombs and separated into developmental stages based on morphological features, according to the protocol described by Jay (1962, 1963). Twelve developmental stages were distinguished. Starting from egg hatching, unsealed larvae were divided into 1-day-old and 2-day-old (L1/2), 3-day-old (L3), 4-day-old (L4) and 6-day-old (L6) worker larvae. The brood in capped cells was divided into spinning*-*stage larvae (L7), prepupae (PP), pupae with white eyes (P1), pupae with white-pink eyes (P2), pupae with pink eyes (P3), pupae with brown eyes and a yellow trunk (P4), and pupae with black eyes and a black body (P5). Newly emerged workers (A) were also studied. The honeybee brood in the same stage from all three colonies was collected. 15 samples contained 3 individuals (one from each colony) representing a given developmental stage (L6–A), except for stages L1/2, L3, and L4, which were represented by samples of 90, 60, and 30 larvae, respectively. The study was conducted on a total of 405 individuals representing stages L6–A, and on 1350, 900, and 450 larvae representing stages L1/2, L3, and L4, respectively. The isolated brood was rinsed in 0.9% NaCl, carefully dried on filter paper, weighed, and immediately frozen in liquid nitrogen and stored at − 70 °C until analysis.

Five samples from each stage were used to analyze the expression of genes encoding glycogen synthase (*gs*), glycogen phosphorylase (*gp*), trehalose-6-phosphate synthase (*tps-1*), cytosolic trehalase (*tre1*) and membrane-bound trehalase (*tre2*). Five other samples were used to measure the protein levels of enzymes glycogen synthase and glycogen phosphorylase. The remaining samples were used to determine the activity of trehalose metabolism enzymes—trehalose synthase, total trehalase activity, and the activity of cytosolic trehalase and membrane-bound trehalase.

### RNA isolation, cDNA synthesis and polymerase chain reaction (PCR)

Total RNA was isolated from the whole body of the brood according to the method described by Chomczyński and Sacchi (1987) with the use of the peqGOLD TriFast reagent (PeqLab, Germany). We treated samples with DNase I (Qiagen) to minimize genomic DNA contamination according to the manufacturer’s instructions. The quantity and purity of isolated RNA were determined by spectrophotometry using the Nano Drop 1000 (Thermo Scientific, Wilmington, USA). cDNA synthesis was carried out according to the instructions provided by the manufacturer of Qiagen kits (Syngen, Poland). Total RNA (2 µg) was reverse transcribed to cDNA using an oligo(dT)_18_ primer. The RT-PCR was carried out at 42 °C for 60 min and at 70 °C for 5 min. The cDNA product with a final volume of 20 µl was stored at − 20 °C until further analysis. PCR was performed with StartWarm Polymerase (A&A Biotechnology, Gdynia, Poland).

### Relative quantification of gene expression

Gene expression was quantified by real-time PCR to compare *tre1, tre2, tps1, gs* and *gp* transcription levels in *A. mellifera* workers. Fold changes in target genes, normalized to *rp49, gpdh, nd5* and relative to the expression levels in endogenous control samples (corresponding to the L1/2 stage), were calculated by the comparative *C*_*t*_ (2^−ΔΔCt^) method (Livak and Schmittgen 2001). Quantitative real-time PCR was performed using the SYBR Green PCR Master Mix (A&A Biotechnology, Gdynia, Poland) according to the manufacturer’s protocol. 20 µl of the reaction solution contained 1 µl of the template (cDNA diluted 1:10), 10 µl of SYBR Green PCR Master Mix, 1 µl of 10 µM of each specific primer, 6.5 µl of water and 0.5 µl of ROX Reference dye II. All samples were analyzed in five replications in the LightCycler system FAST7500 (Applied Biosystem). Melting curves were analyzed to gauge reaction specificity. Mean values ± SD of *C*_*t*_ were used to analyze relative concentrations of mRNA of each gene in every time point with the 99% reaction efficiency, which was obtained earlier according to standard curve (cDNA diluted 1:10, 1:100, 1:1000, 1:1000). The specific primers used in the experiment were designed with Primer3 (http://frodo.wi.mit.edu) (Rozen and Skaletsky 2000) based on gene sequences in the GenBank database (http://www.ncbi.nlm.gov) and the Honeybee Genome Sequencing Consortium (2006) (http://www.hgsc.bcm.tmc.edu). PCR and real-time PCR was performed with the use of Genomed primers (Genomed, Poland) (Table 1). PCR products were evaluated by electrophoresis on 1.5% agarose gel with 0.5 µg of ethidium bromide and were viewed under UV light. Single amplicons were isolated, sequenced and compared with the sequences from the NCBI Blast database. To validate the sequences in *A. mellifera*, the PCR products of *tps-1, tre1, tre2, tpp*, *gs* and *gp* genes in PP and P4 insects were sequenced by Genomed (Poland). Matrix concentration was 729 ± 2.65 ng/µl and primer concentration was 5 pmol/µl.

**Table 1:** Primer sequences in PCR and real time-PCR

Gene	Accession no.	Primers (5′–3′)	PCR product (bp)
*Tps1*	XM_392397	For GAGTTGATCGTAAGAACTTG	210
		Rev ATAGTAACCTGTTCACGATG	
*Tre1*	XP_393963	For GTGGCGTATTACCAGAAAAG′	211
		Rev CCAGATACTTGAGCACCTTC	
*Tre2*	NM 01112671	For GTGGCGTATTACCAGAAAAG′	250
		Rev CCAGATACTTGAGCACCTTC	
*gs*	XM 624704	For TCACTAATCGGTCTAGGATA	230
		Rev AATCGTACAGAACGTCTTAG	
*gp*	XM 623383	For CATCTGATACATAGCCTCAT	150
		Rev TAGTTTCTAGATGGATACGC	
*GAPDH*	M003252010	For GTAGTACAAGAAGCATTGG	210
		Rev TGTATTTAGTGAACGAGAGG′	
*ND5*	GU060470	For TCGAAATGAATAGGATACAG	211
		Rev GGTTGAGATGGTTTAGGATT	
*Rp49*	AF441189	For CGTCATATGTTGCCAACTGGT Rev TGAGCACGTTCAACAATGG3′	210

### Evaluation of glycogen synthase and glycogen phosphorylase proteins

Enzyme-linked immunosorbent assays (ELISAs) were performed to measure the amounts of glycogen synthase and glycogen phosphorylase. The extracts were prepared by grinding 100 mg of a brood sample with 1 ml of phosphate-buffered saline (PBS, pH 7.4) and centrifuging the mixture at 5000×*g* for 10 min at 4 °C. The supernatant was diluted with PBS (pH 7.4) at 1:2 v/v. Microtiter plates (Corning, Sigma) were coated with 100 µl of diluted extracts, incubated for 24 h at 4 °C, and washed three times with 200 ml of PBS containing 0.05% Tween 20. Sites that remained uncoated by the bee antigen were blocked with 1% gelatin in PBS for 2 h, and the plates were washed again with PBS. Primary polyclonal rabbit antibodies against glycogen synthase (SAB 4300648, Sigma) or glycogen phosphorylase (HPA 000962, Sigma) were diluted from 1:1000 to 1:4000 in 1% gelatin in PBS. Then, 100 µl of secondary antibodies (anti-rabbit IgG-peroxidase antibody; A6667, Sigma), diluted 1:5000 with 1% gelatin in PBS, was applied, and the plates were rinsed. Next, 1 ml of peroxidase substrate was added (0.9% hydrogen peroxide in 50 mM citrate–phosphate buffer, pH 5.0, and 0.4 mg of *o*-phenylenediamine) and incubated for 30 min at 37 °C. The reaction was stopped with 5 M HCl, and absorbance was measured with a microplate reader ASYS UVM 340 (Biogenet with Micro Win 2000 software) at 492 nm. ELISA steps were performed at 37 °C with shaking. Negative controls consisted of samples without antibodies against glycogen synthase or glycogen phosphorylase. The results were expressed in nmol enzyme/1 mg of protein. The analyses for each extract were conducted in five replications.

### Determination of enzyme activity

The activity of trehalose-6-phosphate synthase and total trehalase activity was evaluated in brood extracts. The samples were homogenized separately in the Omni TH-02 tissue homogenizer (5000–35,000 rpm; Omni International, USA) in an ice bath with cool 0.9% NaCl (1:10 w/v). One part of the homogenate was centrifuged at 1500*g* for 15 min at 4 °C, and the obtained supernatant was used to determine the activity of trehalose 6-P synthase according to the method of Giaever et al. (1988) and total trehalase activity according to the method of Dahlqvist (1968). The remaining homogenate was centrifuged at 105,000×*g* at 4 °C for 60 min (Beckman SV 55Ti rotor). The resulting supernatant (cytosolic fraction) and precipitate (membrane-bound fraction) were regarded as fractions containing soluble and membrane-bound trehalase (Tatun et al. 2008). The cytosolic fraction and the supernatant of resolved membrane-bound fraction were used to determine the activity of soluble trehalase and membrane-bound trehalase, respectively. Trehalase activity was assessed by measuring the amount of glucose released by the enzyme from trehalose (Dahlqvist 1968). 50 µl of the supernatant and 0.1 ml of 0.5 mM trehalose solution were added to 0.35 ml of 0.2 M phosphate buffer (pH 6.5). The incubation lasted 60 min at 37 °C. Glucose concentration was determined with an enzymatic kit Glucose-OXY (Pointe Scientific, Poland). Enzyme activity was expressed in µmol of glucose per mg of protein. Protein content was measured by the Bradford method (1976).

### Statistical analysis

Differences between mean values of activity and mean values of expression in each bee development stages were analyzed by ANOVA and Tukey’s test in the Statistica 12 program (StatSoft Inc., Tulsa, Oklahoma, USA) at a significance level of *p* < 0.05. The results of the expression of samples, whose averages differ twofold from the relative gene expression of the control, were assumed as statistically significant. The correlations between the activity of the analyzed enzymes and the expression of the corresponding genes were described with the use of Pearson’s correlation coefficient in the Statistica 12 program (StatSoft Inc., Tulsa, Oklahoma, USA).

## Results

### Glycogen metabolism enzymes: glycogen synthase and glycogen phosphorylase

**Fig. 1:**
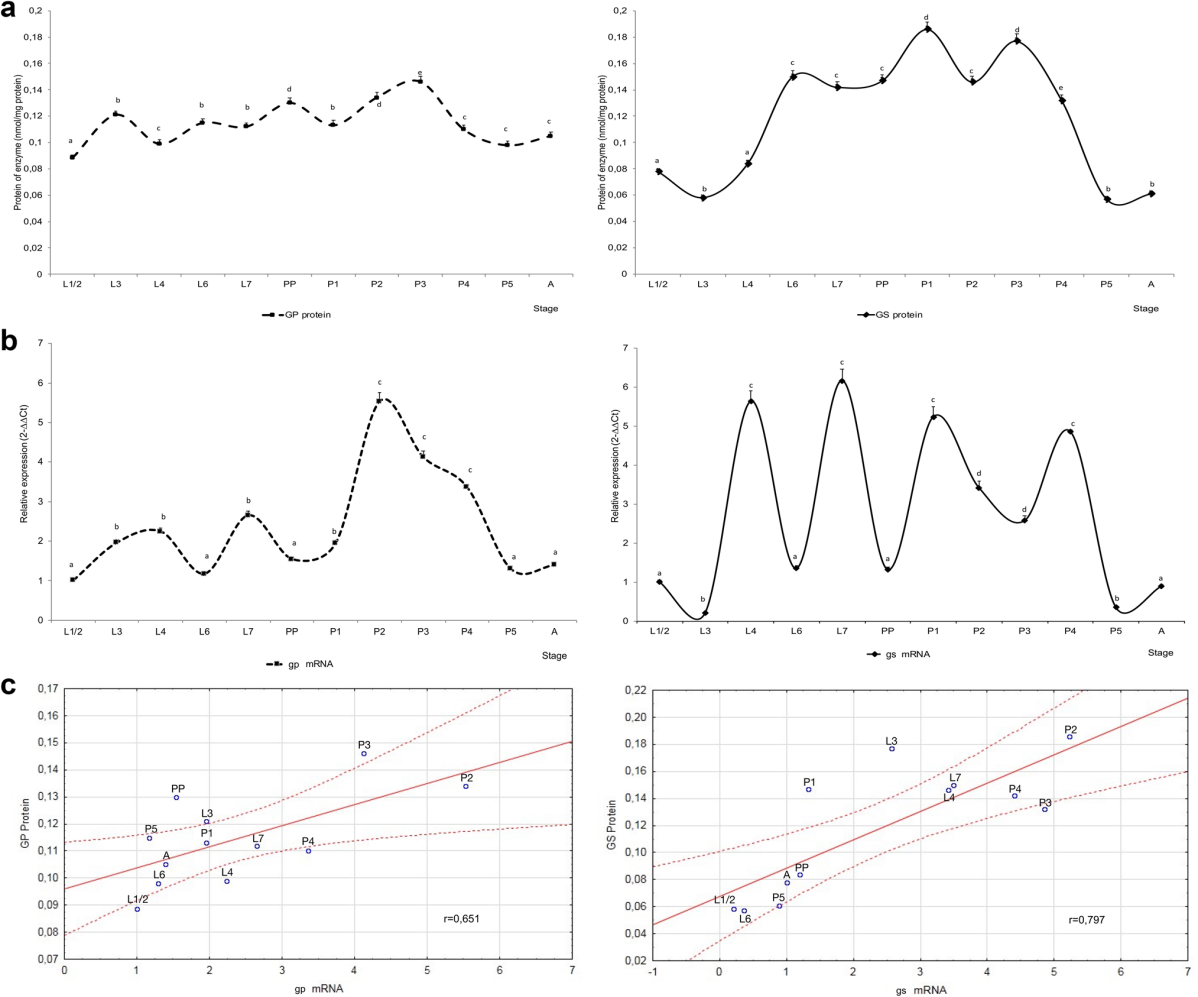
The comparison of gene expression of *gs, gp* and their products during the development of *A. mellifera*. **a** The level of protein glycogen phosphorylase (GP) and glycogen synthase (GS), **b** the expression of mRNA of *gs* and *gp* genes, **c** correlation between protein and gene expression. L1/2—2-day-old larvae, L3—3-day-old larvae, L4—4-day-old larvae, L6—6-day-old larvae, L7—spinning stage larvae, PP—prepupae, P1—pupae with white eyes, P2—pupae with pink eyes, P3—pupae with red eyes, P4—pupae with brown eyes and a yellow trunk, P5—pupae with black eyes and a black body, A—newly emerged workers. Gene expression was normalized by 2^−ΔΔ CT^ method to reference genes *rp49, gpdh, nd5* and an endogenous control sample (L1/L2) where relative quantification (RQ) = 1. The letters above the curves represent significant differences in gene expression or level of protein between means of successive developmental stages

The mRNA of glycogen synthase and glycogen phosphorylase was expressed in all analyzed developmental stages (Fig. 1). The transcription of the *gs* gene was particularly high in L4 and L7 larvae, high in P1 and P4 pupae, and very low in L3 larvae and P5 pupae in comparison with L1/L2. The expression of the *gp* gene was higher in all stages than in L1/2 larvae. The observed differences were significant, excluding in L6 larvae, prepupae, P5 pupae and newly emerged workers (A) (Fig. 1b). During pupation (P1–P4), the shift in *gp* transcripts curve was opposite to *gs* curve. The level of *gp* transcript was higher in P2–P4 pupae than in other stages of development (Fig. 1b). The expression of both glycogen metabolism genes was significantly lower in stages P5 and A (Fig. 1b). The ELISA test confirmed the presence of glycogen synthase and glycogen phosphorylase in the extracts from each developmental stage (Fig. 1a and Suppl. Figure 1). The content of GS protein fluctuated more distinctly than the content of GP protein during development (Fig. 1a). The content of GS protein was lower in L3 larvae, P5 pupae and newly emerged workers (A) than in other stages. In the case of GS, the value of Pearson’s correlation coefficient revealed that transcript levels were strongly correlated with the content of GS proteins (0.797). In the case of GP, transcripts were weakly correlated with GP proteins (0.651) (Fig. 1c).

### Trehalose metabolism enzymes: trehalose 6-P synthase and trehalase

**Fig. 2:**
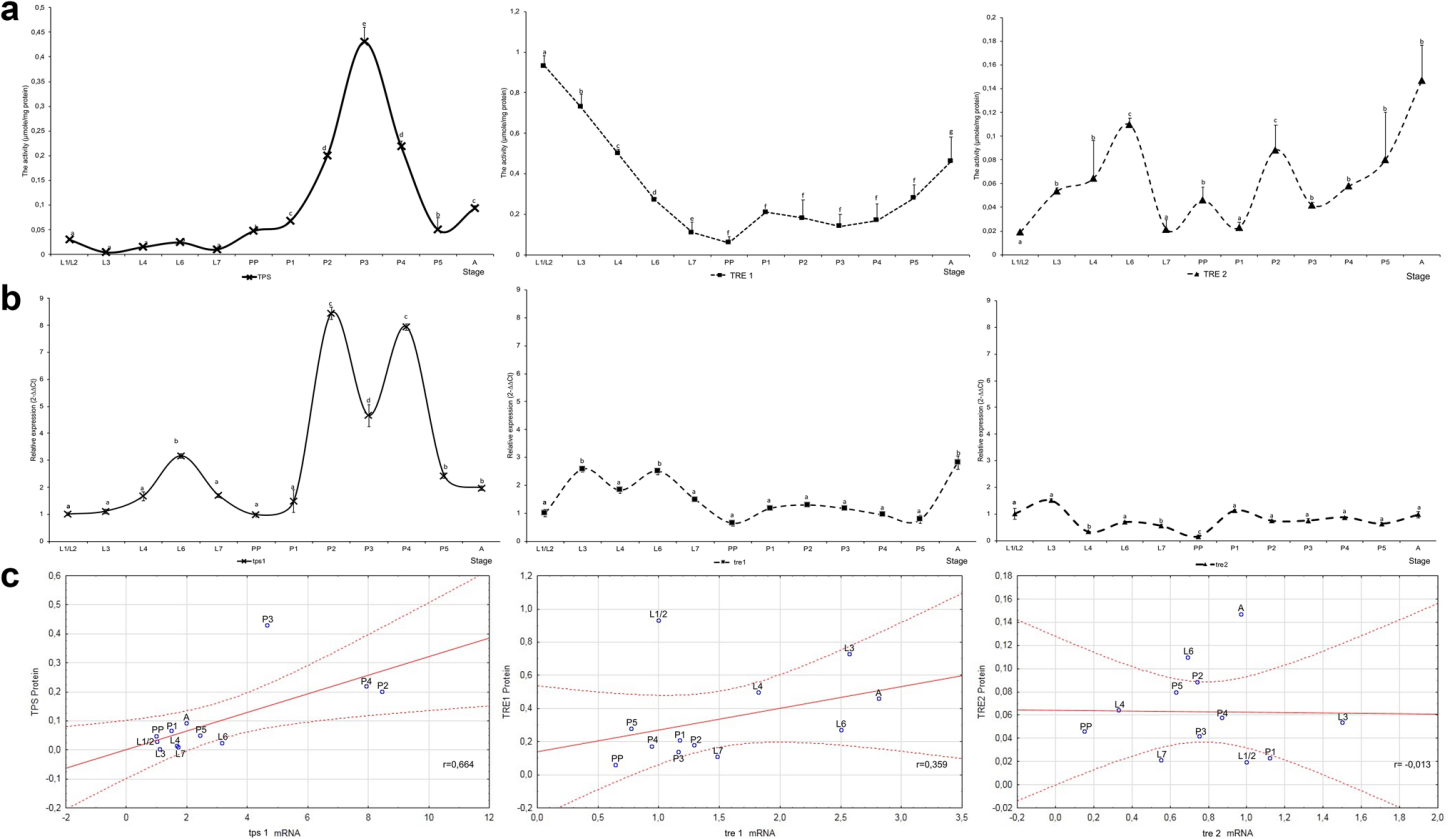
The comparison of gene expression *tps1, tre1, tre2* and their products during the development of *A. mellifera*. **a** The activity of TPS, soluble trehalase (TRE 1) and membrane-bound trehalase (TRE 2), **b** the expression of mRNA of *tps1, tre1, tre2* genes, **c** the correlation between protein and gene expression. Refer to Fig. 1 for explanation

The level of the *tps1* transcript was significantly higher in L6 larvae than other larval stages. During pupation it was higher, excluding PP and P1 stages than in L1/2 larvae (Fig. 2). It was especially visible in P2–P4 pupae, where it was multi-fold higher than in control. Also, newly emerged workers had a significantly higher level of the *tps1* transcript than L1/L2 stage (Fig. 2). The expression of genes encoding soluble trehalase was significantly higher only in L3, L6 larvae and adult workers than in L1/2 larvae. The relative expression of *tre1* gene was higher, compared with *tre2*. The transcript number of *tre2* gene was lowest in L4 larvae and prepupae (Fig. 2b).

The activity of trehalose-6-phosphate synthase was low during larval development and nearly undetectable in L3 and L7 larvae (Fig. 2a). Enzyme activity clearly increased at the onset of pupation and peaked in P3 pupae. At that stage, the activity of T-6-P synthase (0.43 ± 0.07 µmol/mg of protein) was approximately twofold higher than total trehalase activity (0.25 ± 0.01 µmol/mg of protein). The activity of soluble trehalase was higher than the activity of membrane-bound trehalase in all development stages. The changes in soluble trehalase activity were like those noted in total trehalase activity (Figs. 2a vs. 3). In the case of TPS, the value of Pearson’s correlation coefficient revealed that transcript levels were strongly correlated with the content of TPS activity (0.664). In the case of TRE1, transcripts were weaker correlated with TRE1 activity (0.359), but correlation was clear. There was no significant correlation between activity of TRE2 enzyme and the level of TRE2 transcript (− 0.013) (Fig. 2c).

**Fig. 3:**
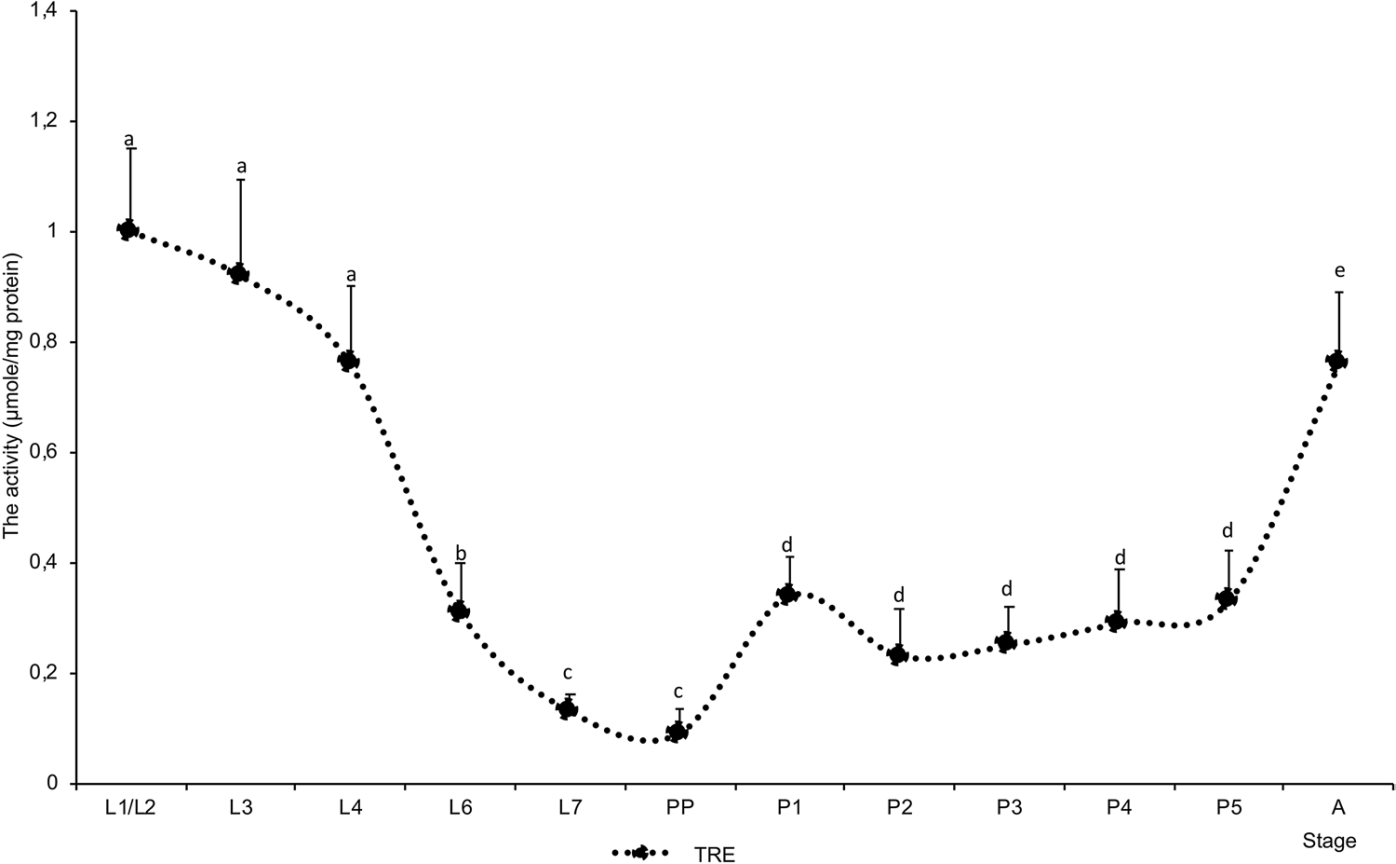
The activity of total trehalase (TRE total) during the development of *A. mellifera*. The letters above the curves represent significant differences (*p* < 0.05) of the TRE total activity between means of successive developmental stage of *A. mellifera*. Refer to Fig. 1 for explanation of development stages

## Discussion

Two key carbohydrates, glycogen and trehalose, guarantee energy homeostasis in insects. Their content is determined by the balance between saccharide synthesis and breakdown processes (Klowden 2007). The rate of carbohydrates metabolism is determined by energy needs and is regulated by hormones. The hormonal regulation of energy homeostasis in mammals has been extensively researched, whereas our knowledge of the relevant processes in insects remains limited. In the investigated insect species, carbohydrate metabolism is hormonally regulated. Hormones such as octopamine, dopamine, juvenile hormone III, ecdysone, adipokinetic/hypotrehalosaemic (Mas-AKH), insulin-like growth factors, allatostatin and corazonin are the main factors responsible for metabolism in insect fat bodies (Lorenz and Gäde 2009; Arrese and Soulages 2010; Soares et al. 2011; Evan et al. 2012). There is almost lack of information about the regulation of sugar metabolism in honeybees, but what is known is that the sugar metabolism mechanism differs across bee subspecies (Lorenz et al. 1999). The hypertrehalosomic hormone controls the metabolism of trehalose and other saccharides in *A. m. ligustica*, but it is not found in *A. m. carnica* (Woodring et al. 2003).

In this study, the genes encoding glycogen synthase and glycogen phosphorylase were expressed throughout development, but their expression levels differed across developmental stages (Fig. 1b). In L4 and L7 stages the relative expression of the *gs* gene was about five- to sixfold higher than in L1/2 larvae, and the expression of the *gp* gene was only around twofold higher than in L1/2 larvae (Fig. 1b). The observed differences indicate that glycogen synthesis proceeds more rapidly than glycogen breakdown in these developmental stages. The L4 stage was interesting because in that stage diet switched from royal jelly to the honey and pollen mix, which is rich in sugars, and excess sugar was stored as glycogen. The high expression of *gs* gene was also observed in L6 larvae, the stage which fed up the last time with remains of food stored up before capping. The above results are confirmation of Farjan’s et al. (2015) results, who observed an increase of the glycogen content from about 3.3 mg/100 mg BW in L1/2 and L3 to 7 mg/100 mg BW in L4–L7 larvae. The expression pattern of *gs* and *gp* genes changed dynamically during pupation (Fig. 1b). The expression of genes encoding the key enzymes of glycogen anabolism and catabolism is controlled hormonally (Hers 1976). Their activities change together by alternating processes of phosphorylation and dephosphorylation of GS and GP proteins (Hers 1976). The above process stabilizes the content of glycogen to cover the energy needs of individual developmental stages. In P1 and P4 pupae, the relative expression of the *gs* gene was much higher in comparison with the *gp* gene. The opposite situation was observed in P5 pupae and newly emerged workers (Fig. 1b). The above findings explain the rate at which glycogen stores are depleted from 5.4 mg/100 mg BW in P2 to 3.0 mg/100 mg BW in A (Farjan et al. 2015). Schmolz et al. (2005) also noted that from capped larval stage the decrease in the content of carbohydrates occurs.

Glycogen phosphorylase has never been isolated from *A. mellifera*, and its structure and properties remain unknown. A comparison of the expression of the glycogen phosphorylase gene with the content of glycogen phosphorylase protein reveals similar changes (*r* = 0.651) in successive developmental stages. (Fig. 1). The content of GS protein noted in this study corresponds to the activity of this enzyme reported earlier in honeybee larvae (Vardanis 1967). In this study, we reported high correlation (*r* = 0.797) between mRNA expression and activity of GS.

Trehalose is the second key carbohydrate in insects. It is synthesized mainly in fat bodies as trehalose-6-phosphate and is accumulated in fat bodies or oocytes because its phosphate form cannot penetrate cell membranes (Elbein et al. 2003). Trehalose has to be dephosphorylated by the second enzyme of the sugar-synthesizing complex—T-6-P phosphatase (TPP). Free trehalose is transported to and from the hemolymph by way of diffusion or by the appropriate transporters (Kanamori et al. 2010). A genome analysis revealed that the honeybees are probably devoid of specific TPP (Kunieda et al. 2006), which is consistent with our observations. Numerous sequencing attempts have been made, with the use of various primers that producted satisfactory results in the other insect, in our study not confirmed specific the expession of the *tpp* gene in any of the analyzed by us developmental stages in workers honeybees. However, phosphatase activity was observed when we used T-6-P as enzyme substrate (data not shown). We suspect that in honeybees, TPP strictly specific for T-6-P would be replaced by phosphatases with broader specificity.

In this study, TPS was expressed and was active throughout brood development (Fig. 2). TPS plays a key role in the development of insects; therefore, its continued presence is not surprising (Tang et al. 2010). Trehalose breakdown into two glucose molecules is catalyzed by specific hydrolases–trehalases. Soluble trehalase (Tre 1) acts within cells, whereas membrane-bound trehalase (Tre 2) hydrolyses hemolymph trehalose and supplies glucose to cells (Mitsumasu et al. 2005). Trehalase has been isolated from many insect species which possessed both forms of the enzyme (Tang et al. 2008; Tatun et al. 2008; Gu et al. 2009; Xie et al. 2013). Similar observations have been made in the honeybee (Lefebvre and Huber 1970; Lee et al. 2007; Mori et al. 2009). In our study, the activity of soluble trehalase was predominant in all developmental stages of the honeybee worker brood (Fig. 2a). The activity of soluble trehalase accounted for 56% of total trehalase activity in P3 pupae to 93% in L1/2 and almost 100% in L7 larvae. Similar results were reported in *Nilaparvata lugens* 5th instar females and adults where soluble trehalase accounted for 75% of total trehalase activity (Gu et al. 2009). Interestingly, the activity of Tre 1 and the expression of the *tre1* gene in *N. lugens* increased after an injection with 20-hydroxyecdysone (20E). The hormone did not affect the expression or the activity of Tre 2 (Gu et al. 2009). The above results indicate that the expression of both trehalase genes is regulated by different mechanisms. These findings suggest that in addition to molting control, ecdysone also influences trehalose metabolism in developing of *N. lugens*. It is not known whether the same mechanism exists in other taxa of insects. Although the effect of ecdysone on the expression of both trehalase genes has not been investigated in the honeybee, our data for P2 (Fig. 2b) were not consistent with maximal ecdysteroid titer which was observed by Zufelato et al. (2000) and Pinto et al. (2002). Moreover, in our study, the changes in the expression of both trehalase genes were similar across the evaluated developmental stages, which may suggest the common control of *thr1* and *thr2* gene expression in the honeybee brood (Fig. 2b). The fluctuations in expression levels of both trehalase genes were more pronounced in the larval stage than during metamorphosis. In the case of *tps* gene expression, the opposite pattern was observed, the fluctuations were higher at pupae stages than larval stages (Fig. 2b). The almost stable content of trehalose during development from L4 to P4 about 1 mg/100 mg of BW, as was stated in our earlier study (Farjan et al. 2015), may be a sign of the balance of expression of genes of the synthesis and breakdown of trehalose. Moreover, the observed changes in the expression of glycogen phosphorylase and TPS genes during pupation were relatively consistent (Figs. 1b vs 2b). Furthermore, the high activity of glycogen phosphorylase during the metamorphosis of bee workers was observed (Żółtowska et al. 2012). The glycogen phosphorylase is responsible for the supply of glucose for trehalose synthesis; therefore, the integrated regulation of the genes encoding both enzymes seems to be justified. The above probably contributes to the maintenance of almost stable trehalose level during starvation period of development (about 1 mg/100 mg BW) at the expense of glycogen metabolism. Glycogen level decreases from 7.4 mg/100 mg BW at early pupae to 3.4 mg at last pupae stage (Farjan et al. 2015).

Our results may also suggest that carbohydrate metabolism genes may be regulated differently during feeding in larval development and during starvation, especially in metamorphosis, but further research is needed to confirm this observation.

## Electronic supplementary material

Below is the link to the electronic supplementary material.

Western blotting of the of glycogen synthase and glycogen phosphorylase. Primary polyclonal rabbit antibodies against glycogen synthase (SAB 4300648, Sigma) or glycogen phosphorylase (HPA 000962, Sigma) were diluted from 1:1000 to 1:4000 in 1% gelatin in PBS. Visualization was made using VECTASTAIN® Elite ABC-Peroxidase Staining Kit (Universal-Mouse/Rabbit IgG) from Vector Laboratories. MW prepared using protein standards. 1- positive control for glycogen synthase; 6 - positive control for glycogen phosphorylase; 4,5 - glycogen synthase, 9 - glycogen phosphorylase; 2, 3, 7, 8 - unpurified samples. (JPG 502 KB)

## References

[CR1] Adeva-Andany MM, González-Lucán M, Donapetry-García C, Fernández-Fernández C, Ameneiros-Rodríguez E (2016). Glycogen metabolism in humans. BBA Clin.

[CR2] Arrese EL, Soulages JL (2010). Insect fat body: energy, metabolism, and regulation. Annu Rev Entomol.

[CR3] Argüelles JC (2014). Why can’t vertebrates synthesize trehalose?. J Mol Evol.

[CR4] Blatt J, Roces F (2001). Heamolymph sugars level in foraging honeybees (*Apis mellifera carnica*) dependence of metabolic rate and in vivo measurement of maximal rates of trehalose synthesis. J Exp Biol.

[CR5] Bradford MM (1976). A rapid and sensitive method for quantitation of microgram quantities of protein utilizing the principle of protein-dye-binding. Anal Biochem.

[CR6] Chang JC, Wu S-M, Tseng Y-Ch, Lee Y-Ch, Baba O, Hwang P-P (2007). Regulation of glycogen metabolism in gills and liver of the euryhaline tilapia (*Oreochromis mossambicus*) during acclimation to seawater. J Exp Biol.

[CR7] Chomczyński P, Sacchi N (1987). Single-step method of RNA isolation by acid guanidinium thiocyanatephenolchloroform extraction. Anal Biochem.

[CR8] Dahlqvist A (1968). Assay of intestinal disaccharidases. Anal Biochem.

[CR9] Dulta PC, Verma LR (1989). Biochemical studies on flight muscles of the genus *Apis*. J Apic Res.

[CR10] Elbein AD, Pan YT, Pastuszak I, Carroll D (2003). New insights on trehalose: a multifunctional molecule. Glycobiology.

[CR11] Evan N, Devaud J-M, Barron AB (2012). General stress responses in the honeybee. Insects.

[CR12] Farjan M, Żółtowska K, Lipiński Z, Łopieńska-Biernat E, Dmitryjuk M (2015). The effect of dietary vitamin C on carbohydrate concentrations and hydrolase activity, during the development of honey bee worker brood. J Apic Sci.

[CR13] Giaever HM, Styrvold OB, Kaasen I, Strøm AR (1988). Biochemical and genetic characterization of osmoregulatory trehalose synthesis in *Escherichia coli*. J Bacteriol.

[CR14] Gu J, Shao Y, Zhang Ch, Liu Z, Zhang Y (2009). Characterization of putative soluble and membrane-bound trehalases in hemipteran insect *Nilaparvata lugens*. J Insect Physiol.

[CR15] Hers HG (1976). The control of glycogen metabolism in the liver. Annu Rev Biochem.

[CR16] Hrassingg N, Crailsheim K (2005). Differences in drone and worker physiology in honeybees (*Apis mellifera*). Apidologie.

[CR17] Jay CS (1962). Colour changes in honeybee pupae. Bee World.

[CR18] Jay CS (1963). The development of honeybees in their cells. J Apic Res.

[CR19] Kanamori Y, Saito A, Hagiwara-Komoda Y, Tanaka D, Mitsumasu K, Kikuta S, Watanabe M, Cornette R, Kikawada T, Okuda T (2010). The trehalose transporter 1 gene sequence is conserved in insects and encodes proteins with different kinetic properties involved in trehalose import into peripheral tissues. Insect Biochem Mol Biol.

[CR20] Kern C, Wolf C, Bender F, Berger M, Noack S, Schmalz S, Ilg T (2012). Trehalose-6-phosphate synthase from the cat flea *Ctenocephalides felis* and *Drosophila melanogaster*: gene identification, cloning, heterologous functional expression and identification of inhibitors by high throughput screening. Insect Mol Biol.

[CR21] Klowden MJ (2007). Integumentary systems. Physiological systems in insect.

[CR22] Kunieda T, Fujiuki T, Kucharski R, Foret S, Ament SA, Toth AL, Ohashi K, Takeuchi H, Kamikouchi A, Kage E, Morioka M, Beye M, Kubo T, Robinson GE, Maleszka R (2006). Carbohydrate metabolism genes and pathways in insects: insights from the honeybee genome. Insect Mol Biol.

[CR23] Lee J-H, Saito S, Mori H, Nishimoto M, Okuyama M, Kim D, Wongchawalit J, Kimura A, Chiba S (2007). Molecular cloning of cDNA for trehalase from the European honeybee, *Apis mellifera* L., and its heterologous expression in *Pichia pastoris*. Biosci Biotechnol Biochem.

[CR24] Lefebvre YA, Huber RE (1970). Solubilization, purification, and properties of trehalase from honeybee *Apis mellifera*.. Arch Biochem Biophys.

[CR25] Livak KJ, Schmittgen TD (2001). Analysis of relative gene expression data using real-time quantitative PCR and the 2^∆∆Ct^ method. Methods.

[CR26] Lorenz MW, Gäde G (2009). Hormonal regulation of energy metabolism in insects as a driving forcefor performance. Integr Comp Biol.

[CR27] Lorenz MW, Kellner R, Woodring J, Hoffmann KH, Gäde G (1999). Hypertrehalosomic peptides in the honeybee (*Apis mellifera*): purification, identification and function. J Insect Physiol.

[CR28] Mitsumasu K, Azuma M, Niimi T, Yamashita O, Yaginuma T (2005). Membrane penetrating trehalase from silkworm *Bombyx mori*. Molecular cloning and localization in larval midgut. Insect Mol Biol.

[CR29] Michelette ERF, Soares AEE (1993). Characterization of pigmental developmental stages in Africanized honeybee workers (*Apis mellifera* L). Apidologie.

[CR30] Mori H, Lee J-H, Okuyama M, Nishimoto M, Ohguchi M, Kim D, Kimura A, Chiba S (2009). Catalytic reaction mechanism based on α-secondary deuterium isotope effects in hydrolysis of trehalose by European honeybee trehalase. Biosci Biotechnol Biochem.

[CR31] Neukirch A (1982). Dependence of the life-span of the honeybee (*Apis mellifera*) upon flight performance and energy consumption. Comp Biochem Physiol B.

[CR32] Panzenböck U, Crailsheim K (1997). Glycogen in honeybee queens, workers and drones (*Apis mellifera carnica* Pollm.). J Insect Physiol.

[CR33] Pinto LZ, Hartfelder K, Gentile Bitondi MM, Sõimes ZLP (2002). Ecdysteroid titers in pupae of highly social bees relate to distinct modes of caste development. J Insect Physiol.

[CR34] Rembold H, Kremer JP, Ulrich G (1980). Characterization of postembryonic developmental stages of female castes of the honeybee, *Apis mellifera* L. Apidologie.

[CR35] Rozen S, Skaletsky H (2000). Primer3 on the WWW for general users and for biologist programmers. Methods Mol Biol.

[CR36] Schmolz E, Kösece F, Lamprecht I (2005). Energetics of honeybee development. Isoperibol and combustion calorimetric investigations. Thermochim Acta.

[CR37] Soares MPM, Silva-Torres FA, Elias-Neto M, Nunes FMF, Simőes ZLP, Bitondi MG (2011). Ecydsteroid-dependent expression of the *tweedle* and *peroxidase* genes during adult cuticle formation in the honeybee, *Apis mellifera*. PLoS One.

[CR38] Stabentheiner A, Pressl H, Papst T, Hrassinngg N, Crailsheim K (2003). Endothermic heat production in honeybee winter clusters. J Exp Biol.

[CR39] Suarez RK (2000). Energy metabolism during insect flight: biochemical design and physiological performance. Physiol Biochem Zool.

[CR40] Tang B, Chen X, Liu Y, Tian H, Liu J, Hu J, Xu W, Zhang W (2008). Characterization and expression patterns of a membrane-bound trehalase from *Spodoptera exigua*. BMC Mol Biol.

[CR41] Tang B, Chen J, Yao Q, Pan Z, Xu W, Wang S, Zhang W (2010). Characterization of a trehalose-6-phosphate synthase gene from *Spodoptera exigua* and its function identification through RNA interference. J Insect Physiol.

[CR42] Tang B, Xu Q, Zou Q, Fang Q (2012). Sequencing and characterization of glycogen synthase and glycogen phosphorylase genes from *Spodoptera exigua* and analysis of their function in starvation and excessive sugar intake. Arch Insect Biochem Physiol B.

[CR43] Tatun N, Singtripop T, Tangjitwitaykul J, Sakurai S (2008). Regulation of soluble and membrane-bound trehalase activity and expression of the enzyme in the larval midgut of the bamboo borer *Omphisa fuscidentalis*. Insect Biochem Mol Biol.

[CR44] Vardanis A (1967). Glycogen synthetase of bee larvae. J Biol Chem.

[CR45] Winston ML (1987). The biology of the honeybee.

[CR46] Wilson WA, Roach PJ, Montero M, Baroja-Fernández E, Muñoz EJ, Eydallin G, Alejandro M, Viale AV, Pozueta-Romero J (2010). Regulation of glycogen metabolism in yeast and bacteria. FEMS Microbiol Rev.

[CR47] Woodring J, Hoffmann KH, Lorenz MW (2003). Identification and function of the hypotrehalosaemic hormone (Mas-AKH) in workers drones and queens of *Apis mellifera ligustica* and *A. m. carnica*. J Apic Res.

[CR48] Xie Y-F, Yang W-J, Wang J-J (2013). Characterization of the cDNA encoding membrane-bound trehalase, its expression and enzyme activity in *Bactrocera dorsalis* (Diptera: Tephritidae). Fla Entomol.

[CR49] Zufelato MS, Bitondi MMG, Simões ZLP, Hartfelder K (2000). The juvenile hormone analog pyriproxyfen affects ecdysteroid-dependent cuticle melanization and shifts the pupal ecdysteroid peak in the honeybee (*Apis mellifera*). Arthropod Struct Dev.

[CR50] Żółtowska K, Lipiński Z, Łopieńska-Biernat E, Farjan M, Dmitryjuk M (2012). The activity of carbohydrate-degrading enzymes in the development of brood and newly emerged workers and drones of the Carniolan honeybee, *Apis mellifera carnica*. J Insect Sci.

