# Supplementary figures and images for: Developmental changes in gene expression and enzyme activities of anabolic and catabolic enzymes for storage carbohydrates in the honeybee, Apis mellifera
Source: Insectes Soc. 2018 Jul 13;65(4):571–80. doi: 10.1007/s00040-018-0648-1 (PMC6208630; doi:10.1007/s00040-018-0648-1)

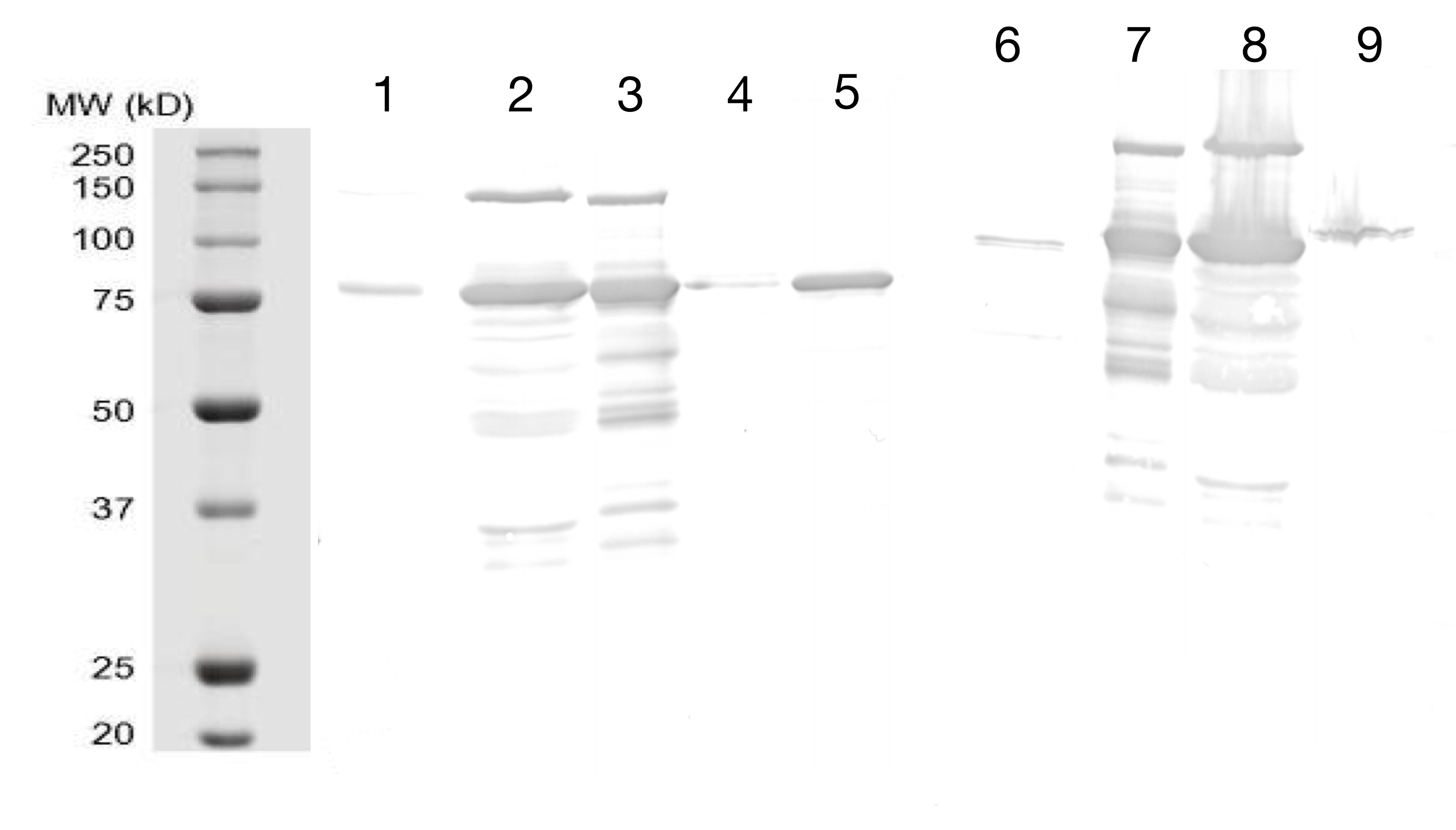

Supplement: Supplementary file 1 — Western blotting of the of glycogen synthase and glycogen phosphorylase. Primary polyclonal rabbit antibodies against glycogen synthase (SAB 4300648, Sigma) or glycogen phosphorylase (HPA 000962, Sigma) were diluted from 1:1000 to 1:4000 in 1% gelatin in PBS. Visualization was made using VECTASTAIN® Elite ABC-Peroxidase Staining Kit (Universal-Mouse/Rabbit IgG) from Vector Laboratories. MW prepared using protein standards. 1- positive control for glycogen synthase; 6 - positive control for glycogen phosphorylase; 4,5 - glycogen synthase, 9 - glycogen phosphorylase; 2, 3, 7, 8 - unpurified samples. (JPG 502 KB) [file 40_2018_648_MOESM1_ESM.jpg]
